# The acceptability of alcohol screening and brief intervention for older adults in community venues

**DOI:** 10.1111/dar.13949

**Published:** 2024-09-16

**Authors:** Jennifer Seddon, Beth Bareham, Eileen Kaner, Barbara Hanratty, Sarah Wadd

**Affiliations:** ^1^ Centre for Psychological Research Oxford Brookes University Oxford UK; ^2^ Population Health Sciences Institute Newcastle University Newcastle UK; ^3^ Substance Misuse and Ageing Research Team University of Bedfordshire Luton UK

**Keywords:** alcohol, implementation, older adults, qualitative, screening and brief intervention

## Abstract

**Background:**

Evidence supports the effectiveness of alcohol brief interventions (ABI) in health‐care settings but the acceptability of conducting ABIs in wider community venues such as supermarkets, hospital atriums and train stations remains unclear. This study examines the acceptability of conducting ABIs for older adults in community settings.

**Method:**

ABIs were conducted in community venues in five sites across the United Kingdom as part of the Drink Wise, Age Well program. ABIs used the Alcohol Use Disorders Identification Test–Consumption to measure alcohol use, with personalised feedback delivered in relation to alcohol intake. Data on age, gender, ethnicity, alcohol use and intention to change drinking was collected. Qualitative interviews to explore the acceptability of delivering ABIs within community venues were conducted with a sub‐set of ABI recipients (*n* = 16) and practitioners (*n* = 12). Data were analysed using Framework Analysis.

**Results:**

A total of 3999 people received an ABI. Fifty‐eight percent of ABI recipients were female. The largest age group was 50–54 years (28%). Almost 80% (*n* = 3180) of ABI recipients were drinking at hazardous levels. Of hazardous drinkers that were asked (*n* = 2726), 40% reported intentions to change their drinking. Qualitative analysis indicted that ABIs conducted in community venues were acceptable and considered to be valuable in raising awareness of alcohol‐related risks.

**Discussion and Conclusions:**

Community venues represent a promising context to engage older people in alcohol intervention, with the potential to lead to reductions in alcohol consumption.

## 1 INTRODUCTION

Hazardous alcohol use is defined as a pattern of alcohol consumption that increases the risk of associated physical, psychological or social harm for the user or others [1]. Increasing numbers of older adults (aged ≥50 years) consume alcohol at hazardous levels. In the United Kingdom, older adults are more likely to exceed recommended low risk guidelines for alcohol use (≤14 standard drink units/week) than any other age group [2], and this trend towards increased alcohol use amongst older adults is mirrored worldwide [3, 4].

Risk of alcohol‐related harm increases with age, as alcohol is metabolised and excreted more slowly in later life [5]. Age‐related physiological changes mean that older adults may experience harm from drinking at levels considered low‐risk for the general population [6]. Alcohol can exacerbate the onset of conditions associated with ageing, such as falls and cognitive impairment; negatively affect the management of common medical conditions in old age, such as type II diabetes and cardiovascular diseases; and interact adversely with medications commonly prescribed for older people [7, 8, 9].

Many older adults are unfamiliar with guidance for low‐risk alcohol intake [10], partly because recommendations have varied over recent decades. Consequently, older adults may not understand or recognise risks relating to drinking or recognise the signs of alcohol dependence [11, 12]. Hazardous drinking can be particularly stigmatised in older populations and feelings of shame, guilt or self‐denial that drinking is excessive or hazardous, can prevent older adults from seeking alcohol‐related support [13, 14, 15, 16]. Most older people drinking at hazardous levels do not require formal alcohol treatment which is typically focused on managing symptoms of physiological dependence. Alcohol screening and brief intervention (ABI) consisting of structured advice or behaviour change support is often most appropriate [17].

Brief interventions for alcohol use are designed to assess the level or pattern of drinking and provide tailored advice to help people reduce their alcohol consumption, and thus reduce the risk of alcohol‐related harm [18]. Following screening, personalised feedback is given on how alcohol use relates to health risks and the array of physical, mental health and social harms associated with risky drinking, and the benefits of reducing intake. Structured advice is provided on how to reduce drinking. This advice is typically delivered using a ‘FRAMES’ approach, a method that emphasises Feedback, Responsibility, Advice, Menu of strategies, Empathy and Self‐efficacy. Interventions are short in duration, typically lasting 5–15 min [18], although ABIs may differ in terms of content, duration and number of sessions.

Primary health care is often seen as the ideal context for the delivery of ABIs: people present with a range of acute and non‐acute conditions, there is frequent patient contact and often an established relationship with the health‐care provider. There is a strong evidence base for the effectiveness of ABIs in primary care populations [18, 19, 20, 21], for both practitioner‐led and digital delivery of ABIs [22]. Effectiveness of ABIs has also been demonstrated amongst older people [23, 24]. However, hazardous alcohol use in older adults is less likely to be identified by health professionals compared to younger people [25, 26], and older adults are less likely to seek specific help for alcohol use [27].

Furthermore, despite evidence for the effectiveness of ABIs in primary care, and inclusion of ABI in protocols for UK National Health Service (NHS) health checks for the over 50s, rates of ABI delivery in this setting are low [28]. The extent to which screening and brief intervention for alcohol use can be conducted in primary care settings is limited by staff workloads, a lack of financial incentives and managerial support, limited alcohol‐related knowledge amongst practitioners and pressures resulting from the COVID‐19 pandemic [29]. Other potential settings for the delivery of ABIs include secondary care and criminal justice settings [30, 31, 32]. Although ABIs are not typically delivered in more general community venues, such as supermarkets and bus stations, these types of locations have shown promise in delivering other health‐care interventions [33, 34]. Future prevention and intervention approaches need to find new ways of identifying and engaging with hazardous older drinkers. Older adults are likely to benefit from more proactive approaches to alcohol brief interventions, but research is needed to establish the feasibility and acceptability of delivering ABIs in more diverse settings.

The implementation of ABIs in community venues, such as supermarkets, hospital atriums and train stations is largely unexplored but may represent a unique opportunity to engage people in conversations about their use of alcohol. This study aimed to investigate the acceptability of conducting ABIs in community venues for people aged 50+.

## 2 METHOD

### 2.1 Study design

A qualitative study to assess the acceptability of ABIs in community venues. ABIs were offered opportunistically in a range of community venues; quantitative data on all ABI respondents were also collected to explore the characteristics of those willing to take part. Acceptability of receiving an ABI in a community venue was assessed via qualitative interview with a sub‐set of ABI recipients and ABI practitioners.

### 2.2 ABI design and setting

Drink Wise Age Well was a community‐based, multi‐intervention, prevention‐to‐treatment program that aimed to reduce alcohol‐related harm in people aged ≥50 years. One of the main aims of the program was to tackle public stigma and provide information and advice in relation to alcohol use [35].

As part of the Drink Wise Age Well program, ABIs were offered opportunistically in five areas across the United Kingdom (Sheffield and Devon, England; Glasgow, Scotland; Cwm Taf, Wales; and Western Health and Social Care Trust Area, Northern Ireland). ABIs were delivered by Drink Wise Age Well alcohol practitioners; including a mix of substance use treatment professionals and peer volunteers. Training to deliver ABIs to older adults included how to screen for hazardous alcohol use, how to provide personalised feedback and when to signpost to sources of further support. Training took place over a period of 2 days and was conducted in‐person, training was led by Drink Wise Age Well staff with a background of working within alcohol treatment services. Intervention fidelity was not formally assessed, although on‐going supervision was available to all ABI practitioners where necessary.

ABIs took place in a variety of community venues, including supermarkets, shopping centres, hospital atriums, health centres, train stations and bowling clubs. Venues were selected based on footfall, visibility, privacy (i.e., if there was space to have a quiet conversation) and the time available for people to engage [36]. Permission was obtained from each community venue to host the ABI stall on their premises. Each ABI stall had clear signage to indicate the focus was on alcohol intake amongst people aged 50+, this precluded the need to screen for age. Workplace environments were avoided due to potential concerns in relation to disclosure of alcohol use in the vicinity of employers. Permission was obtained from host venues in advance. Most members of the public were left to approach the stall on their own terms, although on occasion practitioners asked passers‐by if they wished to take part in ABI screening. ABIs were conducted between 2015 and 2020.

Screening and brief intervention were conducted in‐person using an electronic tablet. The shortened Alcohol Use Disorders Identification Test based on Consumption (AUDIT‐C) was used to screen for hazardous use of alcohol. The AUDIT‐C is a three‐item screening tool, scored on a scale of 0–12. A score of ≥3 for women and ≥4 for men indicates hazardous use of alcohol [37, 38]. The measure has been validated for use with older adults [39]. Recipients were supported to accurately report their alcohol intake (e.g., through use of alcohol unit wheels). Feedback to recipients included information on the screening score, risks associated with their alcohol use including age‐related risks, potential benefits of reducing drinking, and methods to reduce alcohol intake. Feedback was personalised and used infographics to help participants understand their alcohol intake. Health messages were tailored to alcohol use in older age. Feedback was delivered using a ‘FRAMES’ approach [40]. Recipients were signposted to additional sources of support (e.g., to alcohol treatment services) where necessary. Screening and feedback took place at the ABI stall, although practitioners took care to ensure others could not overhear the conversation. The process of screening and brief intervention was designed to take approximately 5 min.

### 2.3 Recruitment

Data from all ABI recipients across sites were included in quantitative analysis. Recipients were self‐selecting members of the public who approached one of the community‐based stalls and consented to take part.

After receiving an ABI, ABI recipients at the Glasgow site (Scotland) were invited to take part in a qualitative interview to assess acceptability of receiving an ABI in a community setting. Those who expressed an interest in taking part were contacted by the research team and provided with an information sheet and consent form. Demographic and alcohol use data were collected at this stage to inform purposive sampling for maximum variation in gender, age, ethnicity, living situation, socio‐economic status (indicated by Index of Multiple Deprivation and [former] occupation), religion, work status, AUDIT‐C risk score and self‐reported health. Recruitment ceased at the point of theoretical sufficiency, where new data added little additional insight into arising issues. All alcohol practitioners at who had been involved in the delivery of ABIs at the Glasgow site (Scotland) were invited to take part in a qualitative interview. Written consent to participate was obtained from all participants prior to interview.

### 2.4 Data collection

Quantitative data for age, gender, ethnicity, alcohol use and AUDIT‐C scores were collected during intervention delivery for all ABI recipients.

Individual semi‐structured qualitative interviews were conducted with ABI recipients within 2 weeks of having received an ABI. Interviews took place by phone or in person, and lasted 15–30 min. Interviews focused on the experience of receiving an ABI in a community venue, including the perceived appropriateness of the setting and the impact of the intervention on understanding and use of alcohol. Drink Wise Age Well alcohol practitioners who had delivered ABIs took part in individual interviews or focus groups, lasting 30–60 min. Interviews focused on experiences of delivering ABIs in community settings, and the perceived benefits and challenges of this approach. All interviews were recorded and transcribed verbatim.

### 2.5 Data analysis

Descriptive analyses of quantitative data for all ABI recipients were conducted using SPSS version 22 statistical software.

Framework analysis was used to support analysis of qualitative data [41]. This involved data familiarisation, developing and refining the analytical framework, indexing data within framework categories, and mapping and interpreting findings. Analysis used the principals of constant comparison to examine intricacies in the data. The perspectives of ABI recipients were triangulated with those of ABI providers to deepen our understanding. While we took a deductive approach to organising our data, our narrative is grounded in the perspectives of ABI recipients and providers (inductive). Analysis focussed on perceptions of the acceptability of alcohol screening and intervention in community venues, engagement and feasibility of conducting ABIs in community venues, and the impact of ABI in community venues for recipients. Data interpretation was informed by Proctor et al.s' [42] framework for evaluation of implementation outcomes; considering applicable concepts of acceptability and appropriateness. NVivo 12 and Microsoft Excel were used for data management, data were analysed by Beth Bareham.

### 2.6 Ethical approval

Ethical approval was granted by the Research Ethics Committees of the University of Bedfordshire (Reference: WADD_IASREC_11_2015) and Newcastle University's Faculty of Medical Sciences (Reference: 1642/9384/2019).

## 3 RESULTS

### 3.1 Quantitative study findings

#### 3.1.1 Sample characteristics

A total of 3999 people received an ABI across the five areas of the United Kingdom (*n* = 628 Sheffield and *n* = 656 Devon, England; *n* = 854 Glasgow, Scotland; *n* = 426 Cwm Taf, Wales; and *n* = 1435 Western Health and Social Care Trust Area, Northern Ireland).

Demographic characteristics of ABI recipients are reported in Table 1. Over half (57.9%, *n* = 2317) of participants were female; the majority of participants were White/White British (90%, *n* = 3602), and the largest participant age group was 50–54 years (27.6%, *n* = 1105).

**TABLE 1:** Alcohol brief interventions recipient characteristics.

Characteristics	All ABI recipients, *n* (%)	Drinking risk level^a^		*p* ^b^
		Hazardous drinkers	Non‐hazardous drinkers	
Gender				0.262
Female	2317 (57.9%)	1864 (58.6%)	453 (56.4%)	
Male	1666 (41.7%)	1316 (41.4%)	350 (43.6%)	
Not disclosed/recorded	16 (0.4%)			
Age, years				0.001
50–54	1105 (27.6%)	912 (28.7%)	188 (23.4%)	OR: 4.85
55–59	705 (17.6%)	595 (18.7%)	108 (13.4%)	OR: 5.51
60–64	674 (16.9%)	545 (17.1%)	126 (15.7%)	OR: 4.33
65–69	663 (16.6%)	503 (15.8%)	159 (19.8%)	OR: 3.16
70–74	484 (12.1%)	368 (11.6%)	114 (14.2%)	OR: 3.23
75+	368 (9.2%)	257 (8.1%)	108 (13.4%)	OR: 2.38
Ethnicity				0.001
White/White British	3602 (90%)	2895 (99%)	697 (95.9%)	OR: 4.15
Asian/Asian British	26 (0.7%)	8 (0.3%)	18 (2.5%)	OR: 0.44
Black/Black British	20 (0.5%)	9 (0.3)	10 (1.4%)	OR: 0.9
Mixed background	12 (0.3%)	10 (0.3%)	2 (0.3%)	OR: 5.0
Other	12 (0.3%)			
Not disclosed/recorded	327 (8.2%)			

Abbreviation: ABI, alcohol brief intervention; OR, odds ratio.

^a^ Data missing for 16 participants in relation to drinking risk level.

^b^ Analysis using ꭓ^2^ test, ORs calculated for likelihood of being a hazardous drinker compared to non‐hazardous drinker for each category.

#### 3.1.2 Alcohol use

Mean AUDIT‐C score was 5.88 for men (SD 3.05, range 0–12), and 5.03 for women (SD 2.82, range 0–12). Screening scores indicate 79.5% of participants (*n* = 3180) scored above the AUDIT‐C threshold for hazardous drinking. The rate of hazardous drinking was similar for both men and women (79.0% and 80.4%, respectively).

A sub‐set of hazardous drinkers were asked questions in relation to whether they had been asked about their use of alcohol by a health, social care or other professional within the last year, and their intention to make changes to drinking. Fifty‐eight percent of participants reported that in the last 12 months, this was the first time they had been asked about their use of alcohol, and 40.6% of participants reported intention to make changes to their drinking following the ABI (Table 2).

**TABLE 2:** Hazardous drinkers: intention to change, previously asked about alcohol use.

	Hazardous drinkers—first time asked about alcohol use in past 12 months by a health, social care or other professional (*n* = 1124)	Hazardous drinkers—Intention to change drinking (*n* = 2726)
*n* (%)		
Yes	653 (58.1%)	1108 (40.6%)
No	405 (36.0%)	1167 (42.8%)
Not sure	66 (5.9%)	451 (16.5%)

### 3.2 Qualitative study findings

#### 3.2.1 Participants

Sixteen participants who received an ABI participated in individual interviews (*n* = 12) or dyadic interviews (*n* = 4). Participant age ranged from 50 to 71 years, with the largest age category 55–64 years (62%, *n* = 10). Participants were predominantly female (69%, *n* = 11). All participants were white British, reflecting the majority of ABI recipients. Seven participants worked full time, six were retired, and three semi‐retired. Participants were from a range of socio‐economic backgrounds as determined by Index of Multiple Deprivation and (former) occupation. AUDIT‐C scores indicated 50% of participants (*n* = 8) were lower‐risk drinkers and 50% (*n* = 8) were hazardous/harmful drinkers. Three‐quarters (75%) of participants had received an ABI in a hospital atrium, while 25% received an ABI in a supermarket foyer.

There were no significant differences in age (ꭓ^2^(25) = 32.37, *p* = 0.15) or gender (ꭓ^2^(1) = 1.57, *p* = 0.29) between participants who took part in qualitative interviews compared to the wider ABI recipient sample, although participants who took part in qualitative interviews were significantly less likely to be a hazardous drinker (ꭓ^2^(1) = 7.27, *p* = 0.03).

Twelve alcohol practitioners took part in either individual interviews (*n* = 5) or focus groups (*n* = 7). The majority of practitioners were female (75%, *n* = 9) and most were white British (*n* = 11). Mean age was 39.6 years (SD 10.2, range 26–57 years). Practitioners had between 9 months and 4 years' experience working with older people to address hazardous alcohol use.

#### 3.2.2 Themes

Findings are detailed under three themes: (i) ABIs in community venues are acceptable; (ii) sensitive delivery is key; and (iii) normalising wider understanding.

#### 3.2.3 ABIs in community venues are acceptable

The majority of ABI recipients reported that receiving an ABI in a community venue was acceptable. Most participants reported feeling comfortable with their alcohol intake and had no reservations about discussing their drinking in a community setting. ABI recipients felt the brief and impromptu engagement regarding their drinking was appropriately pitched to their low level of concern about their intake. This included many people who recognised their drinking was potentially hazardous but felt their drinking was within what they felt to be socially acceptable limits. ABI providers reported that almost all ABIs conducted in community venues were well engaged with and positively received by older people.

‘[Discussing my drinking in public] didn't personally bother me because it's not like you're talking about your sex life, or anything. It's not dead private, what you're drinking’. (Recipient 5, 64‐year‐old woman, hazardous drinker)

‘As long as you explain to people at the beginning what it's about, what we're doing, and what's entailed in it, then, well, they've always been up for it. They've always been up for it, and surprised by it, and interested by it’. (Provider 1)

ABI recipients felt that both of the community locations (i.e., supermarket foyer, hospital atrium) were acceptable for engaging people in conversations about their use of alcohol. Those who had received an ABI in a hospital setting felt this was a particularly appropriate location due to alcohol's relevance to health. Recipients felt the informal setting and relaxed delivery of the ABI created a low‐pressure environment, and some reported that a formal consultation‐style session would be ‘overkill’ and something they would be unlikely to engage with.

‘I think if you had to provide a private space or an office, then people would feel more they were being interrogated, rather than, you know, just generally asking questions about alcohol awareness and stuff like that. I mean, getting dragged round a corner into a private office or something, I would have been more wary and be saying “I'll just not bother.” Going into a private area, it would feel more like I was being screened, rather than asked just some general questions. Once you're in an office, you don't know how long you're going to be there for’. (Recipient 9, 58‐year‐old man, hazardous drinker)

Many ABI recipients did not consider their alcohol use a private topic and were happy to discuss it in a community venue. For recipients who considered alcohol use to be private, most felt adequate privacy was available even in a community setting. However, some ABI recipients felt the setting may not be appropriate to discuss dependent drinking, with concerns regarding confidentiality.

‘For me the setting was fine. I did think that, it's not the kind of setting that, if somebody had a drink problem and they expected their neighbour to come in behind them, that they would have that conversation about drink. I was okay with it, because I don't have a drink problem. I think for most people anonymity would be important, I assume, if you talk about anything that may be considered socially unacceptable’. (Recipient 8, 56‐year‐old woman, lower‐risk drinker)

ABI providers noted that it was difficult to engage people from other ethnic backgrounds in conversations about alcohol use. This suggests that although implementing ABIs in a community space may be acceptable to many people, people from non‐White ethnic backgrounds may be less likely to engage in such initiatives.

‘When you look at stats, it tends to be White Scottish or White British that engage but we do get folk … I'd say religious beliefs can be an issue because in quite a lot of religions, obviously, it's [alcohol's] prohibited. You might get people coming over but, as soon as they see it's about alcohol, they just leave even before you're able to chat to them. I would say that's a barrier. People are drinking within those communities, but in a public setting they're not going to chat to you’. (Provider 3)

Despite the community locations for the delivery of ABIs, the settings were usually regarded as appropriate and acceptable.

#### 3.2.4 Sensitive delivery is key

ABI providers were conscious that alcohol can be a stigmatising and sensitive topic, and that discussion in a non‐health‐care setting must be navigated carefully to prevent distress. Responding in an empathic and sensitive way was essential. Providers also considered it important to have the ability to signpost people to available resources or refer them to other services for additional support. ABI recipients reported that the way ABIs were delivered created a sense of ease that enabled them to disclose and discuss their drinking comfortably. This meant any risk messages were well‐received, and recipients were open to discussing their alcohol use and potential changes in drinking.

‘The staff were very nice, and they had lots of information about things. They weren't too pushy, there was no sort of blame or, “Look what you're drinking, this is really a lot.” I think that's important, not to demonise people, but just for it to be an awareness place, where you're being made more aware, and a bit more education on what it all means to you.’ (Recipient 1, 56‐year‐old woman, hazardous drinker)

However, two ABI recipients had concerns about the ABI provider being younger. For these participants, age was associated with experience and knowledge and an older practitioner was considered to be more appropriate to deliver alcohol‐related information and advice to older people.

‘I don't think they were experienced enough. It's like a teenager telling you how to suck eggs. I'd have felt better with somebody who's been there, done it. I just felt they were a bit young’. (Recipient 12, 63‐year‐old woman, lower‐risk drinker/historic alcohol problems)

#### 3.2.5 Normalising wider understanding

Conducting ABIs in community venues was felt to be an important contribution to addressing alcohol harm in society.

‘I think it's something that's quite useful, I like to see people there talking about alcohol. It's something that I feel quite strongly about, and I think that more people should probably think about alcohol and how much they take. I think it's something that we know about that we don't really understand fully’. (Recipient 1, 56‐year‐old woman, hazardous drinker).

‘That's the first time I've seen it [alcohol screening in public] and we're in our 60s now. I think there should be more of it all the time’. (Recipient 12, 63‐year‐old woman, lower‐risk drinker/historic alcohol problems)

ABI recipients reported that receiving an ABI helped them to better understand their alcohol use, especially in relation to alcohol‐related risks and lower‐risk consumption guidelines. This knowledge helped people to make informed decisions about their drinking. Many recipients reported they had misunderstood the unit content of drinks they consumed prior to the ABI, and had not been familiar with current guidelines for lower risk alcohol use. As a result of the ABI, many recipients reported having reduced their use of alcohol.

‘[the ABI gave me] a bit more awareness, thinking, “Okay, this is maybe a Friday night. How much have I had this week actually?” It might even be 10 o'clock at night and you think, “Oh, I might just have one, the weather's nice,” or whatever. “I might just have one.” There's maybe a bit about actually consciously thinking about how much alcohol you've had that week. If you've had a bit, then I might think twice about, “Well actually, I don't really need that one.”’ (Recipient 11, 53‐year‐old woman, hazardous drinker)

‘I engaged with that [ABI provider] for a while, believing that I don't drink that much, and probably finished the conversation with realising that I drink a bit more than what I probably should drink […] I've made some slight changes. I was drinking a pint and then driving. I hadn't realised that a pint of beer was well over the [Scottish driving] limit […] So I'm not actually drinking now if I go out and play golf. I'll have a pint of soda water and lime, or something. So it has changed my habits a bit’. (Recipient 4, 68‐year‐old man, hazardous drinker)

Beyond the individual level, recipients described some wider effects of ABI. Many remarked on how ABIs provided in community venues helped to normalise discussion of alcohol intake and associated risks, which can be a stigmatised topic in society. A number of recipients described having shared their learning regarding alcohol content of drinks, guidelines and risks associated with excess alcohol intake with peers and family members, suggesting that the learning from this outreach initiative may have had a wider‐reaching impact beyond recipients.

## 4 DISCUSSION

This study demonstrates that ABIs conducted in community venues seem to be acceptable to older adults. A large number of older adults engaged in the intervention. Most felt comfortable discussing their drinking in a community space and welcomed the delivery of ABIs in such settings to raise awareness of lower‐risk drinking. The informal setting was a key strength, with private one‐to‐one consultations in a clinic or consultation room considered too formal. The knowledge gained from participating in ABI motivated many to consider changing their drinking behaviour, with two‐fifths of hazardous drinkers who were asked, reporting the intention to reduce their alcohol intake. The implementation of ABIs in community venues has the potential to reach a diversity of older people drinking at hazardous levels and may inform the implementation of ABIs in more novel settings for the wider population.

Eighty percent of ABI recipients in this study were drinking at hazardous levels, which is higher than rates reported for ABIs conducted in primary care [43], community pharmacy settings [30] or probation services [32]. This finding may reflect age differences in the study samples, as the focus of the current study was on older adults who are more likely to be hazardous drinkers [2]. An alternative explanation relates to the anonymity associated with the community setting. Concerns regarding the recording of drinking behaviour have been highlighted as a barrier to the implementation of ABIs in health contexts, which may prevent people from participating [30]. In contrast, ABIs conducted within community settings are anonymous and data cannot be linked with health‐care records. It is possible that the anonymity associated with conducting ABIs in a community setting may have contributed to a greater proportion of higher‐risk drinkers opting to receive an ABI. This suggests that alcohol‐related interventions conducted in community spaces may be effective in reaching populations that would otherwise be missed from interventions conducted within traditional health‐care settings.

Primary care is seen as the traditional setting for conducting ABIs. However, increased pressures on health‐care services means that the opportunity for alcohol health promotion is much reduced [44]. Utilisation of telehealth services has also increased, especially post‐pandemic [45], which may further reduce the opportunity to conduct alcohol health promotion within primary care settings. Conducting ABIs in more novel settings such as community venues may therefore represent an alternative way to engage people who may be drinking at hazardous levels.

Privacy and confidentiality are key considerations in implementing ABIs in novel settings: lack of privacy has been identified as a salient concern regarding ABIs in pharmacy settings [30, 46]. Despite heightened sensitivity and stigma associated with alcohol‐related discussion in older populations [16], older adults in this study felt adequate privacy was available in a community venue to discuss their drinking. However, concerns were raised that the community setting may not be appropriate for people drinking at high‐risk or dependent levels, which may in part reflect particular stigma associated with dependent drinking [15, 47]. ABIs are not appropriate for those drinking at dependent levels, who often require more intensive treatment. It is important that consideration is given to how to refer people to formal alcohol treatment when implementing ABIs in community venues.

Delivering ABIs in community spaces, such as supermarkets, train stations and shopping centres could help normalise conversations about alcohol use and raise awareness of alcohol‐related risks. This is especially important for older adults who are often not asked about their alcohol use by health professionals [26] and may not understand or may discount the risks relating to alcohol use [16]. Alcohol interventions designed to proactively engage with older people, with tailored messaging and support for alcohol use in older age can help to address this knowledge gap. Older adults found the age‐tailored health messages encompassed in this initiative particularly informative; reflecting findings that older adults are most receptive to age‐specific tailored health messages [11]. Delivering ABIs in community venues represents an opportunity to engage older people who may not have been asked about their use of alcohol before and can help to inform their decision making around alcohol use.

ABI recipients in this study were mainly white British, and the results may not be generalisable to people of other ethnic backgrounds. There is considerable variation in how problem drinking is perceived within different ethnic communities, and although cultural and religious prohibition of alcohol use means rates of alcohol use are lower in many ethnic groups, problem alcohol use and drinking in general may also be concealed within these populations [48]. This can impede help seeking, with minority ethnic groups under‐represented within alcohol treatment [49, 50]. Working with ethnic communities at a local level to raise awareness of alcohol‐related risks has been suggested to increase alcohol health literacy and encourage help seeking [49]. The results of the current study suggest it may not be feasible to engage people from minority ethnic backgrounds in ABIs in community venues, and private healthcare specific settings may instead be more appropriate. Older people with mobility problems may also face exclusion from ABIs in community spaces. More work is needed to better understand how to engage people from marginalised sections of the older population in interventions that target problem drinking [48]. Community engagement approaches [51] show promise in engaging marginalised communities and should be explored in relation to alcohol use interventions.

### 4.1 Strengths and limitations

To our knowledge, this study is the first to explore the acceptability of conducting ABIs in community venues. However, the study has some limitations. The self‐selected nature of the participant sample limits the conclusions we can make about acceptability as people who felt that a community venue was inappropriate were unlikely to have participated. Given the opportunistic approach to screening and the community context, it was not possible to record reasons for not wishing to be screened. Hence, we are not able to establish acceptability as a proportion of an overall eligible population. A larger scale survey may be better suited to ascertain the acceptability of conducting ABIs in community spaces prior to wider implementation. In this study ABIs were conducted in a variety of community venues across the United Kingdom, however, data is not available in relation to the percentage of ABIs conducted within each venue and ABI recipients that took part in qualitative interviews received an ABI in either a hospital atrium or supermarket foyer. All qualitative interviews were conducted with ABI practitioners and recipients from the ABI site in Scotland. There has been much publicity in Scotland in relation to legislation for minimum unit pricing for alcohol [52]; this may have led to increased public awareness and influenced the perceived need and acceptability of interventions for alcohol use.

While this study provides data to indicate large numbers of older adults can be engaged in ABIs in community venues, future research is needed to determine the feasibility of conducting ABIs in these novel settings. There are many challenges to wide‐scale implementation in this context; the nature of the community setting means ABIs cannot be targeted to people who may most benefit, and while the anonymity associated with a community setting may result in increased rates of participation, it prevents any post‐intervention evaluation of alcohol use.

## 5 CONCLUSION

The results of this study demonstrate it is acceptable to deliver alcohol screening and brief intervention in community venues to an older population. ABIs delivered in community venues have the potential to normalise conversations about alcohol and engage older people who may not have been asked about their use of alcohol before. ABIs in community venues could play an important role within prevention and intervention approaches for people drinking alcohol at hazardous levels and have the potential to lead to reductions in alcohol use. Future research should explore the feasibility and effectiveness of ABIs conducted within community spaces.

## AUTHOR CONTRIBUTIONS

Each author certifies that their contribution to this work meets the standards of the International Committee of Medical Journal Editors.

## CONFLICT OF INTEREST STATEMENT

The authors declare no conflict of interest.

## Data Availability

Research data are not shared.
